# Emerging Applications of Metabolomics to Assess the Efficacy of Traditional Chinese Medicines for Treating Type 2 Diabetes Mellitus

**DOI:** 10.3389/fphar.2021.735410

**Published:** 2021-09-17

**Authors:** Yumeng Zhang, Yingbo Yang, Lili Ding, Zhengtao Wang, Ying Xiao, Wei Xiao

**Affiliations:** ^1^ The Ministry of Education (MOE) Key Laboratory for Standardization of Chinese Medicines, Institute of Chinese Materia Medica, Shanghai University of Traditional Chinese Medicine, Shanghai, China; ^2^ Jiangsu Kanion Pharmaceutical Co., Ltd., Lianyungang, China

**Keywords:** traditional Chinese medicine, metabolomics, type 2 diabetes, metabolic regulation, metabolome

## Abstract

Diabetes is a common and complex disease that can exacerbate the complications related to cardiovascular disease, and this is especially true for type 2 diabetes mellitus (T2DM). In addition to the standard pharmacological therapies, T2DM has also been treated with nonconventional regimens such as traditional Chinese medicine (TCM), e.g., herbal medicines and TCM prescriptions, although the mechanisms underlying the therapeutic benefits remain unclear. In this regard, many studies have used metabolomics technology to elucidate the basis for the efficacy of TCM for T2DM. Metabolomics has recently attracted much attention with regard to drug discovery and pharmacologically relevant natural products. In this review, we summarize the application of metabolomics to the assessment of TCM efficacy for treating T2DM. Increasing evidence suggests that the metabolic profile of an individual patient may reflect a specific type of T2DM syndrome, which may provide a new perspective for disease diagnosis. In addition, TCM has proved effective for countering the metabolic disorders related to T2DM, and this may constitute the basis for TCM efficacy. Therefore, further determining how TCM contributes to the reversal of metabolic disorders, such as using network pharmacology or by assessing the contribution of host–gut microbiota interactions, will also provide researchers with new potential targets for pharmacologic-based therapies.

## Introduction

Diabetes is one of the most prevalent chronic diseases worldwide, affecting 463 million people in 2019. It is expected that this number will rise to 700 million by 2045 (Saeedi et al., 2019). Type 2 diabetes mellitus (T2DM) is the most common type of diabetes and is characterized by insulin resistance. Two troublesome effects of T2DM, cardiovascular disease and metabolic disorders, can cause high morbidity and mortality. T2DM is mainly a consequence of environmental and behavioral factors such as a sedentary lifestyle and/or poor dietary choices, which often cause obesity; however, several genetic susceptibilities contribute to disease onset or exacerbate its severity (Zimmet et al., 2001). These factors lead to persistent hyperglycemia and subsequently to decreased insulin sensitivity, which in turn can lead to a spectrum of metabolic abnormalities (Ferrannini et al., 2013). Moreover, persistent disorders of glucose and lipid metabolism may lead to various complications of the microvasculature and macrovasculature, such as stroke, ischemic heart disease, diabetic nephropathy, cognitive dysfunction, and retinopathy (Brownlee, 2001). These conditions have a severe impact on quality of life. Therefore, new and effective measures are needed to prevent the onset of T2DM and improve the management of patients.

Clinically proven medicines for treating T2DM include thiazolidinediones (Thangavel et al., 2017; Nanjan et al., 2018; Oikonomou et al., 2018) as well as sulfonylureas, which stimulate insulin secretion and increase insulin sensitivity to biguanides (Setter et al., 2003). These drugs activate the genes encoding hepatic adenosine monophosphate-activated protein kinase (AMPK) (Wang Q. et al., 2018; Glosse and Föller, 2018; Różańska and Regulska-Ilow, 2018), phosphatidylinositol 3-kinase, and protein kinase B (Akt) (John et al., 2018; Mabhida et al., 2018; Garcia-Galiano et al., 2019). Stimulation of fatty-acid oxidation in an AMPK- and peroxisome proliferator activated receptor-α-dependent manner inhibits interference with c-Jun amino-terminal kinases and insulin action activated by inflammatory cytokines and free fatty acids (Hirosumi et al., 2002; Ferguson et al., 2013). These approaches and candidate drug targets constitute potential means for reducing blood sugar and the incidence of obesity and diabetes symptoms.

Glucose transporter type 4 also plays an important role in maintaining blood glucose homeostasis, which prevents insulin resistance and facilitates the transfer of glucose from blood to the liver through the phosphatidylinositol 3-kinase and Akt signaling pathways. However, the vasculature can be damaged by dysfunction of several metabolic pathways, including the hexamine pathway, the protein kinase C pathway, the glycosylation end-product pathway, and the classic polyol pathway (Hunt and Wolff, 1991; Sheetz and King, 2002).

In addition to the aforementioned drug classes for treating diabetes (i.e., biguanides, thiazolidinediones, and sulfonylureas), alpha-glucosidase inhibitors are also commonly used (Choi and Kim, 2010; Zhou et al., 2013; Ishii et al., 2018). However, these inhibitors often have considerable negative effects, such as drug resistance, hypoglycemia, edema, and weight gain. Advances in the treatment of diabetes have changed the focus from hyperglycemia to controlling glucose metabolism, enhancing the sensitivity of insulin receptors, inhibiting insulin resistance, regulating the non-enzymatic glycosylation of proteins, and downregulating fatty-acid metabolism, among other treatment modalities (Weyer et al., 2001; de Dios et al., 2007). Although many strategies and drugs have been developed for the prevention and treatment of diabetes, current disease management options fall short of complete containment. Current therapies mainly rely on drugs; however, many recently approved diabetes drugs have serious complications, such as hypoglycemia, liver and kidney function damage, and diarrhea (Bekele, 2019). Conventional therapies treat the symptoms of diabetes but do not mitigate metabolic syndrome, which is the major complication of the disease. Traditional Chinese herbal medicines, however, contain a variety of bioactive ingredients that can provide therapeutic benefit for several conditions. For example, *Gynostemma pentaphyllum* (Thunb.) Makino (Jiao-Gu-Lan), *Coptis chinensis* Franch. (Huang-Lian), and *Salvia miltiorrhiza* Bunge (Dan-Shen) can simultaneously enhance insulin sensitivity, reduce visceral fat, and improve hyperlipidemia (Garcia-Galiano et al., 2019). Chinese herbal medicines can also help treat diabetes complications by reversing abnormalities related to blood viscosity, microcirculation, and oxidative stress. Therefore, there is great potential merit in developing new, safe, and effective natural anti-hyperglycemia agents as alternatives to conventional treatments for diabetes and its complications.

The American Dietary Guidelines and the American Diabetes Association (Evert et al., 2013) recommend that diabetes patients as well as healthy individuals eat less refined grains, red meat or processed meat, and sugary drinks to help prevent the onset of T2DM. In addition, in many countries, T2DM patients often take botanical medicines or alternative medicines to potentiate the therapeutic effects of conventional medicines; among these alternatives, traditional Chinese herbal medicines account for a relatively high proportion. Traditional Chinese medicines (TCMs) and their natural bioactive ingredients have a variety of anti-hyperglycemia effects. For example, by eliminating oxygen free-radicals, they help thwart blood hypercoagulability, inhibit the non-enzymatic glycosylation of proteins, inhibit aldose reductase, modulate the metabolism of fats and proteins, and effectively control or delay the onset of diabetes and its complications (Zhang and Jiang, 2012; Lao et al., 2014; Sun et al., 2016; Sharma et al., 2017; Feng et al., 2018). Although an increasing volume of evidence shows that TCMs have a substantial positive impact on treatment of diabetes, research on TCM efficacy remains incomplete.

For many years, researchers have attempted to understand T2DM to formulate interventions and treatment plans to improve patient health (Floegel et al., 2013; Roberts et al., 2014; Zheng et al., 2016). In this regard, high-throughput metabolomics technology has recently begun providing insight into the pathophysiological pathways underlying T2DM (Rhee et al., 2011; Würtz et al., 2012; Padberg et al., 2014).

Metabolomics is the systematic analysis of metabolites in biological samples (Guasch-Ferré et al., 2016), including low-molecular-weight compounds such as amino acids, organic acids, lipids, nucleotides, and sugars. Metabolomics often utilizes approaches based on nuclear magnetic resonance (NMR) and/or various mass spectrometry (MS) techniques, as these technologies not only identify complex metabolic phenotypes but can also be integrated with other “omics” strategies (i.e., transcriptomics, genomics, and proteomics) and bioinformatics data to elucidate potential biological mechanisms and discover clinically relevant diagnostic and prognostic markers of disease risk.

Metabolomics approaches have also been used to study and understand T2DM. A review of recent research revealed that many studies found correlations between T2DM and metabolomics characteristics (Tai et al., 2010). Metabolomics studies can provide insight into disease mechanisms by monitoring differences in metabolite abundance and/or profiles in patients (Cha, 2008; Chan et al., 2009; Carr et al., 2011). Therefore, metabolomics can be used to describe metabolic abnormalities that occur during diabetes progression. Furthermore, metabolomics also has been used for the discovery of disease-related biomarkers (Madsen et al., 2010; Malik et al., 2010), which are commonly used to assess disease severity and the underlying metabolic pathways (Zhang A. H. et al., 2013). Hence, metabolomics can provide a greater understanding of disease pathology and contribute to the development of new treatments (Cruz et al., 2007; Coen et al., 2008). A comprehensive review of T2DM metabolomics is provided by Sas et al. (2015), Gonzalez-Franquesa et al. (2016), and Guasch-Ferré et al. (2016).

## Application of Metabolomics in Studying the Traditional Chinese Medicine Treatment of Type 2 Diabetes Mellitus

For hundreds of years in China, traditional medicine practices have been used to assess disease through personalized diagnosis, whereas modern medical practice is mainly concerned with treating symptoms. In the classic Chinese publication *The Yellow Emperor’s Inner Classic*, the symptoms of “drinking more,” “eating more,” “polyuria,” and “weight loss” that are typical of diabetes are classified as xiao ke zheng, for which TCM has long been a treatment (Ning et al., 2009). According to TCM theory, a disease state reflects an imbalance in any or all of four fundamental aspects—Yin (things related to the physical form of an object), Yang (things related to energy quality), Qi (life-force, which animates the forms of the world), and Xue (dense form of body fluids that have been acted upon and excited by Qi) (Wang et al., 2012) – which are in an unbalanced state when people suffer from a disease. Similarly, patients with T2DM could be classified as having deficiency or excess syndromes, which refer to the organs’ insufficiency or excess in Qi, Xue, Yin, and Yang. A disease or syndrome can be diagnosed via comprehensive consideration of symptoms and signs, including tongue appearance and pulse rate, which can also help determine the cause, location, and nature of the disease as well as the patient’s physical condition, disease status, and prognosis. The diagnosis of any particular disease or syndrome is the most notable attribute of TCM, and all diagnosis and treatment methods are derived from this principle.

Metabolomics technology has been applied to help differentiate among T2DM-related syndromes diagnosed based on TCM practices, and thus metabolomics can assist the standardization of TCM clinical diagnoses, inform our understanding of TCM theory, and provide a basis for modernizing TCM practice. Xu et al. (2012) used high-performance liquid chromatography (HPLC) to analyze the profiles of fatty acids in plasma samples from healthy controls and T2DM patients. They measured levels of 12 fatty acids and assessed four lipid parameters: total cholesterol, triglycerides (TG), and high- and low-density lipoproteins. This analysis documented the levels and profiles of common fatty acids in samples from patients with the three TCM syndromes, noting certain significant differences for the group having Qi deficiency vs. the group having both Qi and Yin deficiencies, and found that the unsaturated fatty acids C18:3 and C20:2 in TG and low-density lipoprotein are potential biomarkers and that C20:5, TG, and high-density lipoprotein are candidate biomarkers for Qi deficiency and damp heat. For Qi and Yin deficiency and damp heat, C14:0, C16:0, C18:1, C18:2, and high-density lipoprotein provide the main classification information, each of which is of great relevance to the diagnosis and treatment of T2DM (Xu et al., 2012). Wu et al. used gas chromatography-mass spectrometry (GC/MS) to carry out metabolic profiling of carbohydrates in urine samples from T2DM patients and healthy control subjects (Wu et al., 2012). They also conducted a comparative analysis of 366 subjects using GC/MS combined with kernel-based orthogonal projections to latent structures (K-OPLS) or subwindow permutation analysis (SPA) to 1) compare urinary carbohydrate profiles between T2DM patients and healthy subjects, 2) determine the relationship between urine carbohydrate levels and TCM syndromes in subjects with T2DM, and 3) determine the characteristics and differences in the distribution of TCM syndromes between patients with mild or severe syndromes. They found that, compared with healthy controls, T2DM patients with deficiency or excess syndromes had substantial abnormalities in glucose metabolism. Patients with deficiency syndrome were older than those with excess syndrome, consistent with TCM theory that Qi, Xue, Yin, and Yang are less prevalent in the elderly than the young. Furthermore, two potential biomarkers, xylose and C4 sugar 2, were discovered in the two syndromes using K-OPLS/SPA and Student’s t-test. The analysis revealed elevated levels of xylose and C4 sugar 2 in patients with excess syndrome compared with those with deficiency syndrome.

**FIGURE 1:**
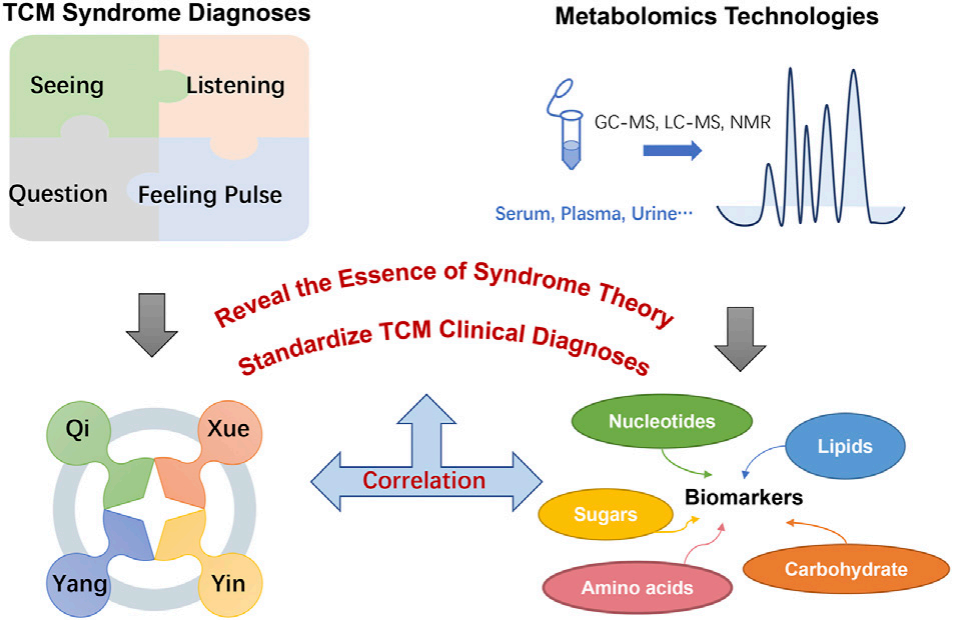
Differentiating TCM syndromes among patients with T2DM.

In summary, syndrome differentiation (Qi, Xue, Yin, and Yang) is the foundation and essence of TCM theory, and the diagnostics mainly depend on overall observation of human symptoms including seeing, listening, questioning, and feeling the pulse rather than “micro” level tests. The metabolic profile is a highly sensitive means of detecting the physiological and pathological changes characteristic of T2DM patients. In addition, a profile can clarify the concept of “syndrome” in the complex physiological system of TCM. Compared with using a single metabolite as a biomarker, using all metabolites to assess human health is more comprehensive and therefore more accurate (Goodacre, 2004; Lu et al., 2009). The research of Wu et al. (2012) demonstrated that the overall application of metabolic profiling in studies of the underlying mechanisms of TCM syndromes is reasonable. Discrimination of different syndromes and the discovery of syndrome-related biomarkers were meaningful for revealing the essence of syndromes. These potential biomarkers reflect dysregulation of metabolism in diabetes patients, which may be helpful for diagnosing diabetes and identifying TCM syndromes, and thus to standardize TCM clinical diagnoses (Figure 1).

## Metabolomics Reveals the Mechanisms Underlying the Efficacy of Traditional Chinese Medicine for Treating Type 2 Diabetes Mellitus

TCM practice has documented success for treatment of T2DM. The guiding principle of TCM is that a variety of traditional Chinese herbal medicines should be used to treat xiao ke zheng, and many classic prescriptions have been passed on through generations of practitioners over thousands of years. The most common Chinese medicine formulations for treating T2DM are Huang-Lian Decoction (HLD) (Pan et al., 2020), Ge-Gen-Jiao-Tai-Wan (GGJTW) (Wang W. et al., 2018), and Qijian mixture (Gao et al., 2018). With continuous practice, experience, and refinement through treatment observations, TCM has formed its own advantages and characteristics relative to modern drugs in terms of regulating glucose and lipid metabolism and countering insulin resistance, having mild yet temporally stable therapeutic effects (Bailey and Day, 1989; Prabhakar and Doble, 2011; Tzeng et al., 2013). For example, Jin-Qi-Jiang-Tang tablets have been proven to improve sugar intake, lipid metabolism, insulin signal transduction, inflammation, and oxidative stress (Liu et al., 2017). Tang-Ning-Tong-Luo formula tablets can reduce liver degeneration, regulate glucose and lipid metabolism, and improve insulin resistance (Cheng et al., 2014). Contemporaneous with treatment, however, patients must adopt healthy dietary habits, emotional and physical exercise habits, and other lifestyle changes to effectively control both blood glucose and blood pressure. To explore the mechanisms underlying these multifaceted interventions, many studies have applied metabolomics methods and produced numerous interesting findings. Table 1 summarizes the various experimental strategies and metabolomics results, including biomarkers and the relevant metabolic pathways.

**TABLE 1:** Information from metabolomics studies of TCM for lowering blood sugar in animal models.

Study ID	Treatment	Model	Control	Size	Sample	Platform	Potential biomarkers for T2DM	Key pathways
Wang W. et al. (2018)	Ge-Gen-Jiao-Tai-Wan	T2DM Sprague-Dawley rats	Control group, diabetes model group	180–200 g	Serum	UHPLC-QTOF/MS	CA, CDCA, TCA, GCA, TCDCA, and taurine↑ Hydroxyacetone, desmosterol, lathosterol, LysoPC(18:1(9Z)), and LysoPE(16:0/0:0) ↓	Bile acid biosynthesis

Gao et al. (2018)	Qijian mixture 0.942 g/kg/day for 8 weeks	Male KKay mice	QJM(H) vs. model	8–9 weeks	Liver	NMR	Leucine, isoleucine, valine, and alanine↑	Valine, leucine, and isoleucine biosynthesis
Cui et al. (2018)	*Scutellariae Radix* (SR), *Coptidis Rhizoma* (CR), and combined extracts 6.3 g/kg for 30 days	T2DM Sprague-Dawley rats	Normal group, model group, metformin group, SR group, CR group, and low dose of combined extracts group	200 ± 20 g	Plasma and urine	UHPLC-QTOF/MS	Cholic acid, deoxycholic acid, xanthosine and deoxyuridine↑	Glycerophospholipid metabolism
							Glycocholic acid, LysoPE, LysoPC, and PC↓	
Pan et al. (2020)	Huang-Lian Decoction (HLD) for 4 weeks	T2DM male Wistar rats	HLD group, model group, and control group	200 ± 20 g	Serum	HPLC-MS	Phenylalanine, l-carnitine, hippuric acid, betaine, and cytosine↑	Glyoxylate and dicarboxylate metabolism, phenylalanine metabolism, and citrate cycle
							Glucose, citrate, and phenylpyruvate↓	

Two main factors regulate insulin secretion: one is nutrients such as glucose, fatty acids, and amino acids; and the other includes neurotransmitters and hormones. Islet cells maintain a certain steady state of secretion in different physiological states through coordination and integration of these two types of factors. T2DM can cause an imbalance among metabolites, such as lipids, carbohydrates, and amino acids. These imbalances can be detected by metabolomics, toward the goal of identifying biomarkers of early-stage disease.

More than 70 years ago, elevated cellular levels of branched-chain amino acids were found to correlate with insulin resistance and diabetes (Luetscher, 1942; Felig et al., 1969; Felig et al., 1974). Metabolomics studies of 74 obese subjects and 67 lean subjects by Newgard et al. (2009) demonstrated that an overabundance of isoleucine, leucine, and valine correlated with the prevalence of coronary artery disease in a cardiac study cohort and also revealed a strong correlation between glutamate and insulin resistance (Newgard et al., 2009); a lower glutamine:glutamic acid ratio predicted an increased risk of developing diabetes (Cheng et al., 2012). Studies showed that an overabundance of free fatty acids (resulting from phospholipid catabolism) may be the main cause of insulin resistance (Taskinen, 2003; Krauss and Siri, 2004). Also, Liu et al. (2010) conducted metabolomics analyses of fatty acids in serum from healthy subjects, T2DM patients, and patients with postprandial hyperglycemia, revealing that elevated levels of palmitic, stearic, oleic, linolenic, and linolenic acids are key indicators of T2DM (Liu et al., 2010).

**FIGURE 2:**
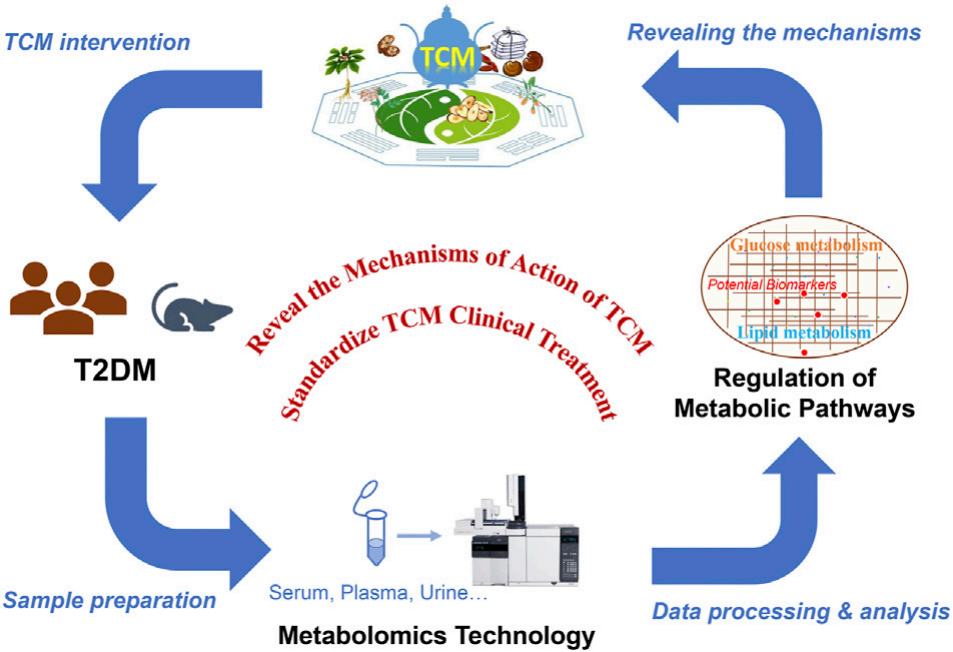
Metabolomics reveals the mechanisms underlying the efficacy of TCM for treating T2DM.

TCM has a long history of treating T2DM, but the underlying mechanism remains unknown. In agreement with the holistic concept of TCM, metabolomics has shown great potential in evaluating efficacy of TCM for treating T2DM. By analyzing endogenous metabolites of small molecules in samples from T2DM patients and examining the metabolic status of the organism, metabolomics can reveal the metabolic changes and the underlying mechanism involved in the pathogenesis of diabetic complications and the efficacy of TCM for treating the disease. Generally, TCM treatment may significantly change some metabolic disorders (e.g., glucose or lipid metabolism) associated with T2DM, promoting metabolic network reorganization through restoring of key metabolites and metabolic pathways, which may be the main mechanism providing the basis of TCM treatment of T2DM. In addition, the discovery of additional diabetes biomarkers would have breakthrough significance for the development of diabetes drugs (Figure 2). Below, we summarize several commonly used herbal medicines for diabetes treatment and the identification of potential T2DM biomarkers.

Georgi root, the dry root of *Scutellaria baicalensis* Georgi, has several biological activities such as anti-inflammation (Yang et al., 2018), anti-cancer (Cheng et al., 2018), and anti-oxidation (Zhang et al., 2011) and has been used to treat various diseases. The major bioactive components of Georgi root are flavonoids such as baicalin, wogonoside, baicalein, and wogonin. Accumulating research with T2DM patients has shown that baicalin can mitigate insulin resistance (Yang et al., 2012; Song et al., 2013) and suppress gluconeogenesis (Wang et al., 2017). *Coptidis Rhizoma*, the dried rhizome of *Coptis chinensis* Franch. (Huang-Lian), contains numerous alkaloids – the major ones being berberine, coptisine, and palmatine—and has been used to treat diarrhea for thousands of years in China. Modern studies have shown that berberine can also substantially lower blood glucose and promote insulin secretion (Zhang et al., 2014; Neag et al., 2018; Xie et al., 2018; Belwal et al., 2020), but the mechanism underlying its role in relieving T2DM symptoms remains unknown. However, potential biomarkers and related pathways have been identified via comparative analysis of plasma and urine metabolomics data for normal and T2DM rats (Cui et al., 2018). Several studies identified eight potential T2DM-related metabolites, including cholic, deoxycholic, and glycocholic acids, the latter of which may inhibit inflammation via TGR5 activation (Agarwal et al., 2018). Cholic and deoxycholic acids block glycocholic acid binding to TGR5. Three other compounds—phosphatidylcholines, lysophosphatidylcholines, and lysophosphatidylethanolamines—are mainly involved in the pathogenesis of inflammation and metabolic diseases such as diabetes. Another compound, xanthosine, promotes the production of uric acid, which is involved in several diabetes complications. The study demonstrated that extracts of Georgi root and *Coptidis Rhizoma* can have a substantial therapeutic effect for patients with T2DM by modulating the activities of both pro-inflammatory cytokines and enzymes related to glucose metabolism (Cui et al., 2018).

HLD was first described in the classic Chinese medicine volume *Treatise on Febrile Diseases* (Shang-Han-Lun), mainly with regard to treatment of Yin and Qi deficiencies. Two other ancient books, *Physician’s Record* (Ming-Yi-Bie-Lu) and *Explanation of Materia Medica* (Ben-Cao-Jing-Ji-Zhu), also promoted the use of prescriptions containing *Coptis chinensis* Franch. For more than 1,500 years, these prescriptions were found to benefit patients with T2DM and were first described in Ming-Yi-Bie-Lu during the Wei Jin Dynasty (An and Cui, 2008). HLD is composed of *Coptis chinensis* Franch. (Huang-Lian); *Rhizoma Zingiberis*, the fresh rhizome of *Zingiber officinale* Roscoe (Gan-Jiang); *Glycyrrhiza uralensis* Fisch. ex DC. (Gan-Cao); *Cinnamomum verum* J.Presl (Gui-Zhi); *Panax ginseng* C. A. Mey (Ren-Shen); *Pinellia ternata* (Thunb.) Makino (Ban-Xia); and *Ziziphus jujube* Mill. (Da-Zao). Pan et al. (2020) reported that HLD can effectively regulate the levels of numerous compounds in various metabolic pathways that are affected in patients with T2DM (Pan et al., 2020). Using HPLC-MS, they discovered that HLD regulates the production of several compounds – such as cytosine, phenylalanine, glucose, l-carnitine, phenylpyruvate, betaine, citrate, and hippuric acid—that are involved in dicarboxylate and glyoxylate metabolism, phenylalanine metabolism, and the tricarboxylic acid cycle. All these metabolites are related to glucose and lipid metabolism, which are affected by HLD.

GGJTW is composed of *Pueraria montana* var. *lobata* (Willd.) Maesen & S.M.Almeida ex Sanjappa & Predeep (Ge-Gen), *Coptis chinensis* Franch. (Huang-Lian), and *Cinnamomum verum* J.Presl (Rou-Gui). Jiao-tai-wan, which comprises solely the latter two herbs, was first mentioned as a treatment for insomnia in the classic medical work “Han-Shi-Yi-Tong” of the Ming Dynasty. Puerarin is one of the main bioactive ingredients in *Pueraria montana* var. *lobata* (Willd.) Maesen & S.M.Almeida ex Sanjappa & Predeep, and has been used therapeutically for diabetes and its complications (Wu et al., 2013). Puerarin can mitigate insulin resistance (Chen et al., 2018) and protect islet cells (Rojas et al., 2018). GGJTW is used to treat diabetes in China owing to its potent anti-hyperglycemia effect. However, little was known about the underlying metabolic mechanism until 37 potential biomarkers were detected using a metabolomics approach based on ultra-HPLC coupled with quantitative time-of-flight (QTOF) MS. The majority of these biomarkers participate in the biosynthesis of primary bile acids involving increased production of taurine and cholic, chenodeoxycholic, taurocholic, glycocholic, and taurochenodeoxycholic acids. The observed significant changes in the levels of these metabolites demonstrated the anti-hyperglycemia effect of GGJTW on diabetic rats and its potential metabolic mechanism (Wang W. et al., 2018).

The Qijian mixture contains four herbs, each of which is commonly used in TCM to treat diabetes: *Astragalus mongholicus* Bunge (Huang-Qi); *Pueraria montana* var. *lobata* (Willd.) Maesen & S.M.Almeida ex Sanjappa & Predeep (Ge-Gen); *Ramulus euonymi*, an extract of *Euonymus alatus* (Thunb.) Sieb. (Gui-Jian-Yu); and *Coptis chinensis* Franch. (Huang-Lian) (Gao et al., 2018). *Astragalus membranaceus* was formally described by Carl Linnaeus in his book *Plant Species* in 1753, and it has a long history in China with wide-ranging clinical applications. *Astragalus mongholicus* Bunge lowers blood lipids and blood sugar, eliminates edema and oxidative stress (Nozaki et al., 2017), and has a broad range of pharmacological effects on diabetes (Liao et al., 2017). For T2DM in particular, it has a potent therapeutic benefit (He et al., 2018). For example, Gao et al. (2018) used 1H-NMR to assess how the Qijian mixture affects the metabolomic profiles of various liver cell types in samples from T2DM patients and to explore the pharmacodynamics. The major metabolites affected by the Qijian mixture follow: isoleucine, choline, leucine, valine, sn-glycero-3-phosphocholine, citrate, myo-inositol, glycerol, anserine, trimethylamine n-oxide, glutarate, lactate, trimethylamine n-oxide, alanine, glucose, acetate, homoserine, inosine, 3-hydroxybutyrate, glutathione, taurine, niacinamide, xanthine, glycine, and adenine (Table 1). Four metabolites—valine, alanine, isoleucine, and leucine—potentially mediate the anti-hyperglycemia effect. The results demonstrated that the Qijian mixture can regulate amino-acid metabolism by decreasing the levels of these four amino acids. The catabolism of leucine, isoleucine, and valine is linked to insulin sensitivity, i.e., an increase in the levels of these three free-amino-acids in serum promotes insulin sensitivity. The biosynthetic pathways for alanine, aspartate, and glutamate contribute to the pathogenesis of metabolic syndrome in T2DM. Thus, four pathways—branched-chain fatty-acid metabolism and the alanine, aspartate, and glutamate biosynthesis pathways – are closely associated with T2DM, confirming the therapeutic potential of the Qijian mixture for T2DM.

## Further Perspectives in Metabolomics Studies of Traditional Chinese Medicine for Treating Type 2 Diabetes Mellitus

### Network Pharmacology for Discovery of New Drugs for Treating Type 2 Diabetes Mellitus With Traditional Chinese Medicine

Metabolomics can show the reversal of metabolic disorders caused by TCM treatment, whereas network pharmacology can help to determine how TCM contributes to these changes (Zhang G. B. et al., 2013; Mao et al., 2017). Network pharmacology employs “omics” technologies to detect different molecules (genes, enzymes, and metabolites) and annotate them by comparing with specific databases. Specifically, TCM network pharmacology is used to investigate TCM from a systems perspective and at the molecular level, updating the research paradigm from the current “one target, one drug” mode to a new “network target, multi-components” mode. This method is specialized to prioritize disease-associated genes, to predict the target profiles and pharmacological actions of herbal compounds, to reveal drug–gene–disease co-module associations, to screen synergistic multi-compounds from herbal medicines in a high-throughput manner, and to interpret the combinatorial rules and network regulation effects of herbal medicines (Li and Zhang, 2013). The network-based method has been demonstrated effective to identify the key ingredients in TCM formulations and predict potential molecular targets in T2DM. Below, we summarize some typical examples.

The classic TCM formulation Ge-Gen-Qin-Lian Decoction (GGQLD) has good clinical effects on T2DM which consists of four herbs: *Pueraria montana* var. *lobata* (Willd.) Maesen & S.M.Almeida ex Sanjappa & Predeep (Ge-Gen), *Scutellaria baicalensis* Georgi (Huang-Qin), *Coptis chinensis* Franch. (Huang-Lian), and *Glycyrrhiza glabra* L. (Gan-Cao) (Tong et al., 2011; Zhang C. H. et al., 2013). A network pharmacology approach was employed to determine the potential antidiabetic ingredients from the GGQLD formula. Further *in vitro* antidiabetic trials demonstrated that a predicted antidiabetic ingredient from Ge-Gen, 4-hydroxymephenytoin, can increase the insulin secretion in RIN-5F cells and improve insulin resistance in 3T3-L1 adipocytes (Li et al., 2014), indicating that the network pharmacology strategy is a powerful means for identifying bioactive ingredients and mechanisms of action for TCM herbal formulae.

Compound Lian-Ge granules are composed of *Coptis chinensis* Franch. (Huang-Lian), *Pueraria montana* var. *lobata* (Willd.) Maesen & S.M.Almeida ex Sanjappa & Predeep (Ge-Gen), *Salvia miltiorrhiza* Bunge (Dan-Shen), and *Thlaspi arvense* L. (Hai-Zao), resveratrol, and taurine Xue et al. (2019) used network pharmacology methods to predict the 24 bioactive components of compound Lian-Ge granules for lowering blood sugar, including berberine, puerarin, danshinolic acid A, and sinigrin, for which nine targets and 111 metabolic pathways were implicated (Xue et al., 2019). Using network pharmacology methods and technologies, this study predicted the hypoglycemic targets of main active ingredients in Lian-Ge granules and revealed their action modes of in multiple pathways, providing a theoretical basis and a clue for exploration of the hypoglycemic mechanism of compound Lian-Ge granules.

As a traditional Chinese herbal medicine, *Astragalus mongholicus* Bunge is widely used clinically to treat diabetes. Li et al. (2019) discovered 13 key T2DM targets and found that *A. membranaceus* can treat T2DM by upregulating the activity of casein kinase, by ensuring the normal regulation of lipid metabolism, and by enhancing insulin resistance, thereby upregulating insulin signaling. Several key targets were randomly selected for quantitative real-time PCR validation, and the results indicated that the analysis of network pharmacology was robust and targets identified via this process were worthy of validation (Li et al., 2019).

In summary, these studies confirmed the feasibility of combining metabolomics and network pharmacology to study the metabolic pathways involved in T2DM that are affected by TCM. In future studies, this combined approach may be a potentially powerful tool for discovery of bioactive components of TCM and elucidation of their action mechanisms for treating T2DM.

### Role of Gut Microbiota in Treating Type 2 Diabetes Mellitus

In recent decades, the gut microbiome has emerged as an integral aspect of the efficacy of TCM, and increasing evidence supports a role for the microbiome in the treatment of T2DM. Subjects of T2DM can be distinguished based on a reduced number of gut Clostridiales bacteria (*Roseburia* species and *Faecalibacterium prausnitzii*), which produce the short-chain fatty acid butyrate (Qin et al., 2012; Karlsson et al., 2013). Short-chain fatty acids are one of the most important metabolites produced by the gut microbiota, because these metabolites can affect host glucose–insulin homeostasis by modulating the activities of G protein-coupled receptors, promoting the secretion of various hormones, and stimulating the vagus nerve. Finally, another important metabolite of lipopolysaccharide-producing, Gram-negative bacteria has been identified as a trigger of insulin resistance (Cani et al., 2007).

Gegen Qinlian Decoction has long been used to treat common metabolic diseases such as T2DM. Xu et al. found that treatment with both berberine and Gegen Qinlian Decoction significantly altered the overall profile of the gut microbiome and enriched many butyrate-producing bacteria, including *Faecalibacterium* and *Roseburia*, thereby reducing intestinal inflammation and lowering blood glucose level (Xu et al., 2020). *Sophora flavescens* Aiton (Ku-Shen) is a well-known Chinese herbal medicine that has been used to combat T2DM (Jung et al., 2008). It was recently reported that flavonoid compounds in the ethyl-acetate extract of *Sophora flavescens* Aiton can regulate the metabolism of lipids, carbohydrates, and especially amino acids in patients with T2DM by mediating the host–microbe metabolic axis (Shao et al., 2020). Therefore, it is feasible to identify potential gut microbiota-related targets in T2DM patients for patient-specific treatment with TCM.

## Conclusion and Perspective

**FIGURE 3:**
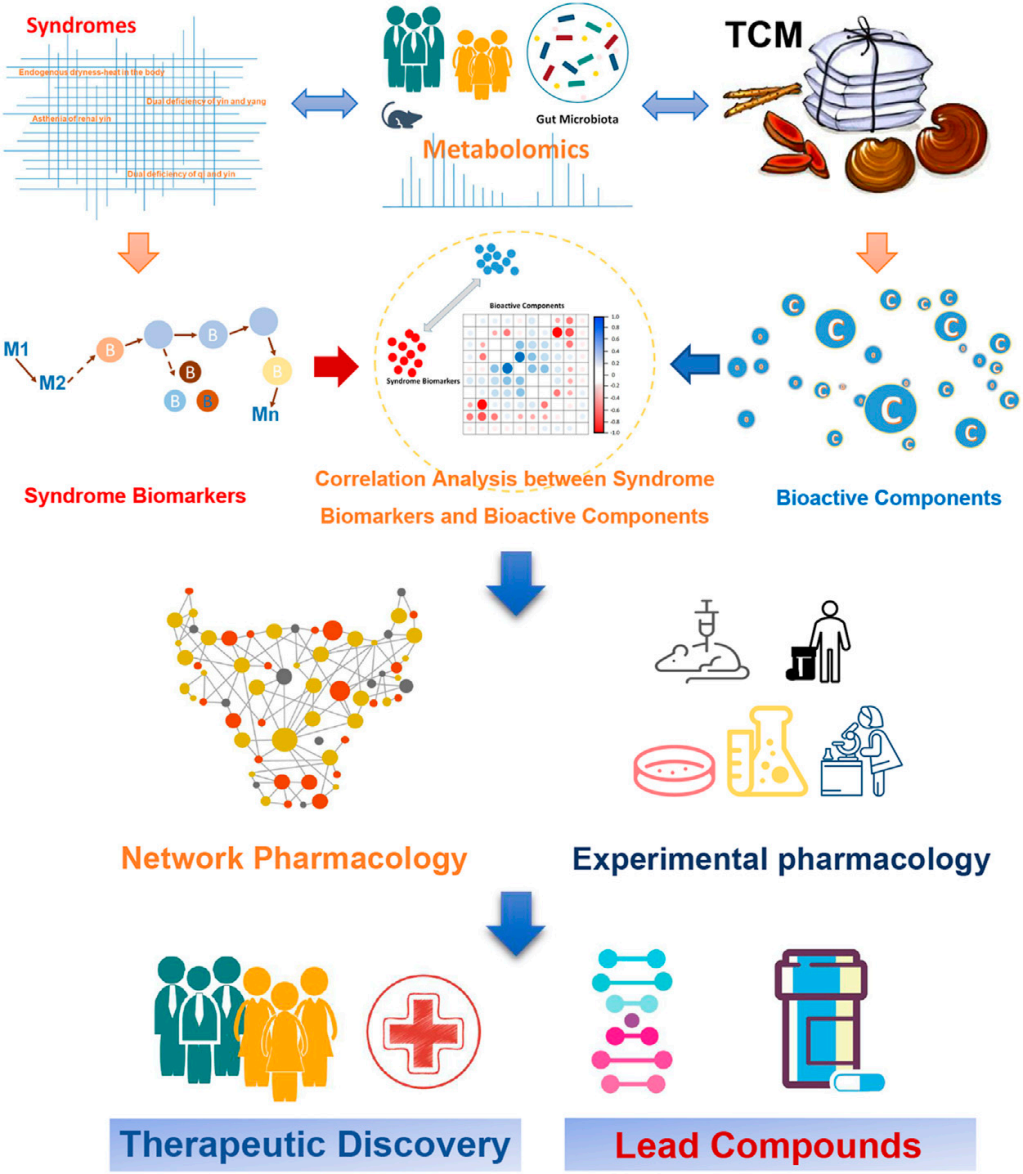
Application of metabolomics to assess the efficacy of TCM for treating T2DM, for the purpose of developing new anti-diabetes therapeutics.

T2DM is a major global health problem, which is treated as “Xiaoke” in the TCM system, and the related herbal medicines have been used over thousands of years. However, due to its complexity, it is very difficult to decipher the scientific basis and systematic features of TCM for treating T2DM. In the systemic context, metabolomics has a convergence with TCM, therefore it could provide useful tools for uncovering the essence of TCM. Metabolomics can be used to systematically explore the pathophysiology of T2DM and elucidate the overall molecular mechanism underlying the known positive effects of treatment with TCM. Previous studies have shown that metabolomics can help distinguish the various T2DM syndromes. In addition, metabolic disorders in diabetes patients can be mitigated by treatment with TCM, reflecting TCM’s anti-hyperglycemia effect. Research has shown that network pharmacology methods, in combination with experimental pharmacology, can be further used to identify the bioactive ingredients in TCM and their targets, which could inform the development of new therapies for T2DM (Figure 3). The emerging application of metabolomics to elucidate the pathways underlying the efficacy of TCM for T2DM will assist efforts to identify new T2DM biomarkers and develop novel anti-diabetes therapeutics. Moreover, the integration of metabolomics and TCM shows promise in bridging the gap between Chinese and Western medicine and helping to interpret the essence of TCM, and thus perhaps enabling a revolution for future health care.

It is noteworthy that although a considerable number of potential biomarkers for T2DM have been identified via integration of metabolomic approaches into TCM, there are still some limitations. Due to the lack of a universal analysis platform and a mature and consistent operation plan, most of them have not been analyzed in a standard fashion, and few have been clinically validated. Therefore, it is necessary to establish a comprehensive and complete operation method by expanding the research content of metabolic tissue, improving technical methods, and perfecting the database. Furthermore, it is required to strengthen the combined application of metabolomics technology with other multi-omics, such as genomics and proteomics, to make up for the shortcomings of metabolomics itself, so as to achieve comprehensive and efficient clinical application from research content, and accelerate the standardization and modernization of TCM for treating T2DM.

## Abbreviations

Akt, protein kinase B; AMPK, adenosine monophosphate-activated protein kinase; GC/MS, gas chromatography-mass spectrometry; GGJTW, Ge-Gen-Jiao-Tai-Wan; GGQLD, Ge-Gen-Qin-Lian Decoction; HLD, Huang Lian Decoction; HPLC, high-performance liquid chromatography; K-OPLS, gas chromatography-mass spectrometry; MS, mass spectrometry; NMR, nuclear magnetic resonance; SPA, subwindow permutation analysis; T2DM, type 2 diabetes mellitus; TCM, traditional Chinese medicine; TG, triglycerides.
